# A machine learning approach to predict the glaucoma filtration surgery outcome

**DOI:** 10.1038/s41598-023-44659-6

**Published:** 2023-10-24

**Authors:** Luca Agnifili, Michele Figus, Annamaria Porreca, Lorenza Brescia, Matteo Sacchi, Giuseppe Covello, Chiara Posarelli, Marta Di Nicola, Rodolfo Mastropasqua, Paolo Nucci, Leonardo Mastropasqua

**Affiliations:** 1 Department of Medicine and Ageing Science, Ophthalmology Clinic, University “G. D’Annunzio” of Chieti-Pescara, Via Dei Vestini, 66100 Chieti, CH Italy; 2 Ophthalmology Unit, Department of Surgical, Medical, Molecular Pathology and Critical Care Medicine, University of Pisa, Pisa, Italy; 3 Department of Medical, Oral and Biotechnological Sciences, Laboratory of Biostatistics, University “G. d’Annunzio” Chieti-Pescara, Chieti, Italy; 4 University Eye Clinic, San Giuseppe Hospital, IRCCS Multimedica, Milan, Italy; 5 Department of Neuroscience, Imaging and Clinical Science, “G. d’Annunzio” University of Chieti-Pescara, Chieti, Italy

**Keywords:** Diseases, Medical research, Risk factors

## Abstract

This study aimed at predicting the filtration surgery (FS) outcome using a machine learning (ML) approach. 102 glaucomatous patients undergoing FS were enrolled and underwent ocular surface clinical tests (OSCTs), determination of surgical site-related biometric parameters (SSPs) and conjunctival vascularization. Break-up-time, Schirmer test I, corneal fluorescein staining, Meibomian gland expressibility; conjunctival hyperemia, upper bulbar conjunctiva area of exposure, limbus to superior eyelid distance; and conjunctival epithelial and stromal (CET, CST) thickness and reflectivity (ECR, SCR) at AS-OCT were considered. Successful FS required a 30% baseline intraocular pressure reduction, with values ≤ 18 mmHg with or without medications. The classification tree (CT) was the ML algorithm used to analyze data. At the twelfth month, FS was successful in 60.8% of cases, whereas failed in 39.2%. At the variable importance ranking, CST and SCR were the predictors with the greater relative importance to the CART tree construction, followed by age. CET and ECR showed less relative importance, whereas OSCTs and SSPs were not important features. Within the CT, CST turned out the most important variable for discriminating success from failure, followed by SCR and age, with cut-off values of 75 µm, 169 on gray scale, and 62 years, respectively. The ROC curve for the classifier showed an AUC of 0.784 (0.692–0.860). In this ML approach, CT analysis found that conjunctival stroma thickness and reflectivity, along with age, can predict the FS outcome with good accuracy. A pre-operative thick and hyper-reflective stroma, and a younger age increase the risk of FS failure.

## Introduction

Despite the recent diffusion of novel and less invasive surgical procedures, filtration surgery (FS) is still the most diffuse and effective approach to lower intraocular pressure (IOP) in patients with uncontrolled glaucoma^1^. Nevertheless, the success rates of FS tend to reduce over time, with the possibility to reach and maintain long-term low IOP values limited by various pre-, intra-, or post-surgical determinants^2^.

Among determinants advocated to increase the risk of surgical failure, African ancestry, younger age, advanced stage of glaucoma, high pre-operative IOP, the presence of concomitant ocular or systemic diseases and a severe form of glaucoma therapy-related ocular surface disease (GTOSD) are considered some of the most important^3^. Thus, the pre-operative risk assessment, especially focused on the ocular surface evaluation, represents the most crucial step to define the risk profile for failure as it may supplement the decision-making processes of clinicians. The risk assessment may in fact guide surgeons in adopting the most appropriate surgical procedure, and implementing personalized pre-, intra-, and post-operative treatment plans to improve the outcomes^4^.

To date, though several predictors have been proposed over the years, the objective assessment of the risk of surgical failure remains a major challenge. Recently, machine learning (ML) gained immense attention in health sciences and has been widely tested in different types of surgery. Studies conducted in patients undergoing neurosurgery, arthroscopic hip preservation or colorectal surgery, or radiofrequency catheter ablation for atrial fibrillation, found that ML models outperform clinical systems for predicting the risk of surgical failure^5–8^. However, to date, the scientific literature that investigated the utility of ML in glaucoma surgery is very poor. In fact, ML has been used to predict the need for surgical intervention in patients with a progressive disease in one study, and the risk of FS failure in patients’ candidates to trabeculectomy in another study^9,10^. In this latter study, Banna et al. compared four ML predictive models to predict the one-year success of FS^10^. They found that the random forest model, which integrated demographic and systemic data with disease-related features, offered value in predicting the FS outcome, with an accuracy of 0.68 and an AUC of 0.74.

The CART (Classification And Regression Tree) method is a supervised ML algorithm for constructing a data-driven prediction model. The model is obtained by recursively partitioning the data space and fitting a simple prediction model within each partition. As a result, the partitioning can be represented graphically as a decision tree. In particular, Classification Trees are designed for dependent variables that take a finite number of unordered values (i.e., binary outcome)^11^. By using CT, the present study aimed to predict the surgical outcome in patients undergoing trabeculectomy by considering demographic characteristics, glaucoma-related clinical data, and ocular surface features.

## Results

The demographic characteristics of groups and baseline features are reported in Table 1.

**Table 1:** Descriptive statistics and OR [95%CI = Confidence interval] for the logistic univariate analysis for demographic and clinical data of groups at baseline.

Variables	Success *N* = *62*	Failure *N* = *40*	OR [95%CI]	p-value
Sex, n (%)				0.990
Females	25 (40.3%)	17 (42.5%)	Ref	
Males	37 (59.7%)	23 (57.5%)	0.91 [0.41;2.07]	
Age, years median [q1; q3]	74.00 [60.20;80.00]	71.00 [62.80;78.20]	1.00 [0.98;1.03]	0.923
MD, dB median [q1; q3]	− 7.46 [− 15.79; − 22.00]	− 9.59 [− 15.04; − 18.20]	1.00 [0.98; 1.02]	0.581
IOP, mmHg median [q1; q3]	25.00 [22.20;28.10]	23.0 0[21.00;26.00]	1.02 [0.89;1.01]	0.426
Duration of therapy, mo median [q1; q3]	10.00 [5.00;15.00]	10.00 [5.00;72.00]	1.00 [1.00;1.01]	0.353
N° of medications	3.00 [3.00;4.00]	3.00 [3.00;5.00]	1.04 [0.76;1.41]	0.888

The p-value results from univariate logistic regression models.

*MD* mean defect, *dB* decibel, *IOP* intraocular pressure, *mo* months.

At baseline, no significant differences were found for IOP, the number of IOP-lowering medications, and the stage of glaucoma between success and failure. The 87.9% of patients were pseudophakic before trabeculectomy, whereas 5.2% of subjects underwent phaco surgery after trabeculectomy. In none of the cases were reported significant intra- or post-operative complications. At twelve months, surgery was successful in 60.8% of cases, whereas failed in 39.2%. The last follow-up IOP was 15.7 ± 1.3 mmHg for successful cases, and 24.1 ± 2.5 mmHg for failed cases (p < 0.05), whereas the mean number of post-operative bleb manipulating procedures were significantly higher in failure (2.1 ± 0.5) compared to success (0.9 ± 0.3).

According to standard descriptive statistics, all considered variables did not differ between success and failure (Table 2).

**Table 2:** Descriptive statistics and OR [95%CI = Confidence interval] for the logistic univariate analysis for clinical and AS-OCT ocular surface variables.

Variables	Success *N* = *62*	Failure *N* = *40*	OR [95%CI]	p-value
BUT, s Median [q1; q3]	8.00 [5.00;10.00]	7.00 [3.00;10.00]	1.07 [0.94;1.22]	0.362
STI, mm Median [q1; q3]	7.50 [4.00;16.50]	9.00 [6.00;15.00]	1.01 [0.95;1.05]	0.655
OSDI	19.0 [12.5;32.5]	24.0 [15.8;41.6]	0.98 [0.96;1.01]	0.171
CFS, n (%)				0.291
0	13 (21.0%)	11 (27.5%)	Ref	
1	19 (30.6%)	6 (15.0%)	2.60 [0.77;9.50]	
2	16 (25.8%)	9 (22.5%)	1.49 [0.47;4.86]	
3	7 (11.3%)	5 (12.5%)	1.17 [0.28;5.15]	
MGE, n (%)				0.414
0	11 (17.7%)	7 (17.5%)	Ref	
1	22 (35.5%)	10 (25.0%)	1.39 [0.40;4.77]	
2	17 (27.4%)	9 (22.5%)	1.20 [0.33;4.28]	
3	6 (9.68%)	9 (22.5%)	0.44 [0.10;1.79]	
BCH, n (%)				0.453
0	5 (8.06%)	3 (7.50%)	Ref	
1	29 (46.8%)	16 (40.0%)	1.10 [0.19;5.33]	
2	22 (35.5%)	13 (32.5%)	1.03 [0.17;5.15]	
3	5 (8.06%)	4 (10.0%)	0.77 [0.09;5.78]	
CET, µm Median [q1; q3]	60.00 [50.20;76.00]	59.70 [46.30;73.90]	0.99 [0.97;1.01]	0.243
CST, µm Median [q1; q3]	110.00 [92.90;137.00]	91.9 [71.4;126]	0.99 [0.99;1.00]	0.054
ECR Median [q1; q3]	116.00 [99.30;132.00]	112.00 [97.70;127.00]	1.00 [0.98;1.01]	0.768
SCR Median [q1; q3]	187.00 [177.00;203.00]	185.00 [166.00;203.000]	0.99 [0.97;1.01]	0.353
AEC, µm^2^ Median [q1; q3]	325.00 [202.00;440.00]	309.00 [252.00;469.00]	1.00 [1.00;1.00]	0.563
LED, µm Median [q1; q3]	12.70 [8.84;15.20]	12.20 [10.40;14.30]	1.01 [0.91;1.12]	0.679

The p-value results from univariate logistic regression models.

*BUT* break up time, *STI* Schirmer test I, *OSDI* ocular surface disease index, *CFS* corneal fluorescein staining, *MGE* Meibomian gland expressibility, *BCH* bulbar conjunctival hyperemia, *CET* conjunctival epithelial thickness, *CST* conjunctival stromal thickness, *ECR* epithelial conjunctiva reflectivity, *SCR* stromal conjunctiva reflectivity, *AEC* area of exposure of the bulbar conjunctiva, *LED* limbus eyelid margin distance.

The agreement between the observers for each of the OCT parameters are reported in Table 3.

**Table 3:** Mean of OCT measurements for each observer and ICC (intra-class correlation coefficient) for inter-observer agreement (SD = standard deviation).

Measurements	Observer 1	Observer 2	ICC (95%CI)
CET, µm, mean (SD)	63.51(19.73)	64.15(19.44)	0.998 (0.996 to 0.998)
CST, µm, mean (SD)	112.23(43.24)	112.12(44.14)	0.975 (0.962 to 0.983)
ECR, mean (SD)	112.05(24.17)	113.93(27.08)	0.922 (0.892 to 0.951)
SCR, mean (SD)	185.35(25.32)	186.08(27.33)	0.954 (0.931 to 0.969)

**Figure 1:**
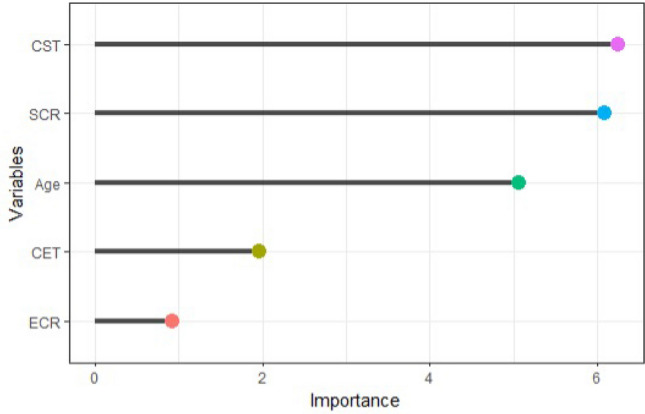
Relative variable importance ranking (VIR) of features for the classification tree (CT) analysis.

When looking at the variable importance ranking (VIR) of considered variables, we found that CST, followed by SCR, was the predictor variable of surgical outcome with greater relative importance to the construction of the final tree (Fig. 1).

Age presented relative intermediate importance, whereas CET and ECR were the predictor variables with the lowest level of relative importance. Conversely, ocular surface clinical test and surgical site-derived parameters were not able in predicting the outcome.

**Figure 2:**
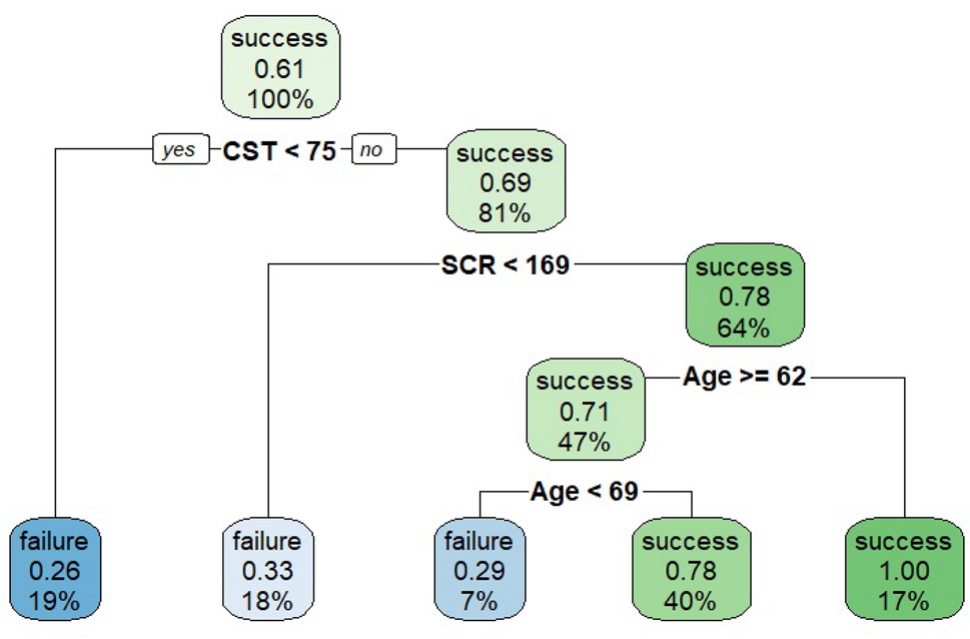
Classification tree (CT) for surgical success versus failure showing data on 102 patients for model building.

When CT was further grown, we found that CST turned out the most important variable for discriminating success from failure, followed by SCR and age (Fig. 2).

In our sample, starting from a situation in which 61% of patients belong to success and 39% to failure, the maximum decrease in node impurity was obtained by splitting on CST. As it is a continuum, the tree finds the optimal cut-off value, thus bringing it back to a binary variable.

**Figure 3:**
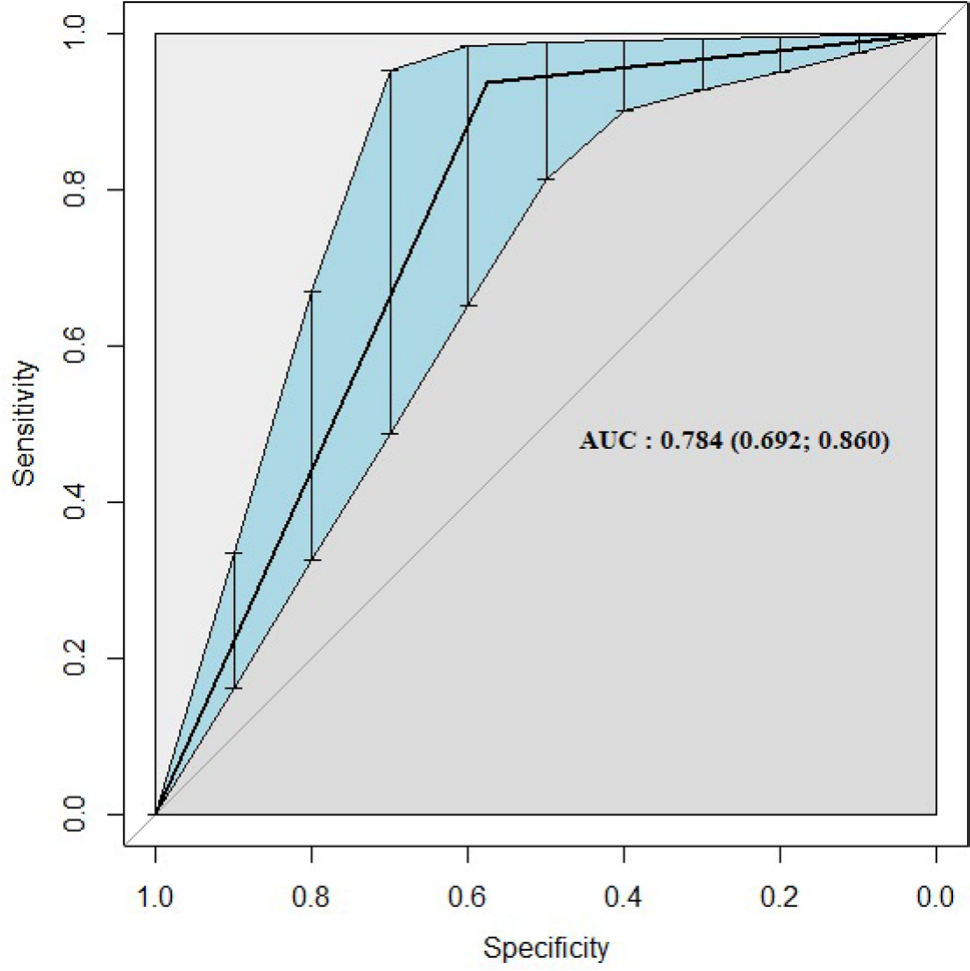
The ROC curve for the classification tree (CT) classificatory and AUC (95% CI = Confidence interval).

When analyzing the tree, one may note that whether CST is lower than 75 µm, patients will be splitted to the left having a 26% of probability having a failure (19% of the sample in the terminal node), whereas for CST values higher than or equal to 75 µm (81% of the sample) patients will be splitted to the right. In this case the dataset is splitted based on the second important variable, the SCR, with a cut-off value of 169 on the gray scale. When considering this variable, if a patient has an SCR value lower than 169, there is a failure for 18% of the remaining sample. Following the path of the tree, in case SCR is higher than or equal to 169, the right side of this second split considers the third important variable, which is the age: for patients aged less than 62 years, we found a success probability equal to 100% (17% of the sample). For patients with CST ≥ 75, SCR ≥ 169 and age less than 69 years, the probability of success is 29%, whereas, for patients with age greater or equal to 69 years, the probability of success is 78%. Figure 3 reports the ROC curve for the classifier showing an AUC 0.784 (0.692 – 0.860), which indicates a good diagnostic efficacy.

## Discussion

Among the numerous aspects gravitating around the field of glaucoma surgery, the need to predict the risk of failure before the procedure still represents one of the most relevant. As known, besides demographic and disease-related variables, the ocular surface impairment represents the most important determinant for the outcome^1–4^. In the present study, we utilized an ML algorithm (CART) to predict the risk of failure in patients undergoing filtration surgery taking into consideration: (i) demographic, (ii) glaucoma-, and (iii) ocular surface-related features.

In general, we didn’t find significant differences between successes and failures when applying a standard statistical approach, with an OR close to one for all variables of the three groups of features. Conversely, when adopting the CT algorithm, we found that some structural features of the conjunctiva, the stromal thickness and reflectivity, were the important predictors of the surgical outcome, followed by age.

In detail, patients undergoing surgery with a thicker and hyper-reflective stroma, and with an age younger than 69 years, showed a higher risk of failure one year after trabeculectomy. In the VIR, the thickness and reflectivity of the conjunctival epithelium were the variables with the lowest importance, whereas none of the ocular surface related-clinical features were significant predictors of the surgical outcome. These results generally agree with a previous AS-OCT study of Mastropasqua and coworkers, which found that CST and SCR were predictors of the twelve months surgical outcome, with both studies being concordant on the importance of the conjunctival stroma as a potential source of predictive biomarkers^12^. However, when analyzing these two studies in detail, they in part differed since Mastropasqua et al. found that a thinner and hyper-reflective conjunctival stroma were predictors of failure. Therefore, data of stromal reflectivity agree whereas data or thickness are in contrast. This inconsistency may certainly depend on the nature of the analysis performed, but also on the characteristics of samples, which show a different numerosity (102 vs 67 patients), a different mean age (74 vs 63 years) and could have had a different story for medical therapy.

As demonstrated, medical therapy for glaucoma impairs conjunctival stroma promoting inflammatory cells infiltration, fibroblast activation, and increasing collagen deposition^3,13–15^. In relation to the duration of therapy and regimens adopted over the years, these changes affect tissue composition and, thus, stromal thickness and reflectivity can appear strongly modified before surgery. The effects that medical therapy produces on conjunctival layers depend on the class of medications, especially for the stroma. While the epithelium thickens regardless of the type of medication, stroma thins in case of therapy regimens include prostaglandin analogs (which promote the extracellular matrix degradation), whereas thickens when regimens do not include PGAs^13,15–17^. Thus, generally, a large of patients undergoing surgery (who are under a maximal medical therapy), may show a thick epithelium and a thin stroma.

Besides stromal features, we found that also age represents an important predictor of the surgical outcome. This result is in line with literature that demonstrated that younger age increases the likelihood of surgical failure^3^. This is a remarkable aspect, since some studies found a significant relationship between stromal thickness and age, with CST decreasing throughout the entire lifetime^18^. Thus, younger subjects have a thicker conjunctival stroma that may predispose to unfavorable responses to incisional surgeries. Therefore, conjunctiva and age appear strictly interdependent between them.

In our analysis the epithelial changes of the conjunctiva were less important predictors of the surgical outcome; this seems reasonable since the resistance induced by the epithelium on the aqueous humor resorption through the bleb-wall is lower than that produced by stroma, which is rich in collagen fibers. Of note, our model did not find the ocular surface test and surgical site-related features as significant predictors of the outcome. This could depend on the fact that clinical test is in part operator and environmental conditions dependent, as well as the hyperemia level determination and, thus, they could not objectively reflect the deep structural changes of the conjunctiva. Moreover, evidence of correlations between ocular surface tests and stromal features of the conjunctiva in glaucoma is not available. For surgical site-related features, which indicate the surface available for surgical maneuvers, one may hypothesize that the technical execution of surgery, when performed without complications, does not affect the final outcomes.

To date, just one study evaluated the utility of ML algorithms in predicting the trabeculectomy outcome^10^. In this study, Banna and coworkers compared four different ML algorithms reporting that the random forest was the top performing model, with an accuracy of 0.68 and AUC of 0.74. The authors considered systemic, demographic, and ocular data observing that the most important predicting features were the central corneal thickness, the age, the body mass index, and the visual acuity. In our study, the CT algorithm performed slightly better (AUC of 0.784) with the advantage to have included structural features of the conjunctiva, which are more directly related to surgery compared to systemic or other glaucoma-related variables.

CT may have some advantages over other ML algorithms. First, CT is highly intuitive and easy to understand. Second, the rules implemented by CT can be displayed in a flow chart-like manner, allowing the model’s predictions to be easily explained. This induces confidence in the stakeholders and provides detailed information about what is happening and why it is happening. Thus, unlike other algorithms, CT is easy to interpret, visualize and comprehend. Third, the CT algorithm is non-parametric. Thus, recursive partitioning does not make any distributional assumptions about the modelled variables and accounts for multi-level interactions among variables. Outliers in the input variables have no meaningful effect on CT^19^.

The present study has some limitations. First, conjunctival measures obtained by AS-OCT could vary among operators. However, the previous study of Mastropasqua et al. found a high level of inter-observer agreement for conjunctival thickness parameters^19^. Second, we used a single ML algorithm to analyze data; thus, we cannot state whether other algorithms would have performed better, would have found additional predictors other than thickness, reflectivity, and age, or would have found important predictors completely different. Further studies comparing CT with other ML algorithms on all variables we investigated, are needed. Finally, given that ML algorithms could work on a high number of variables, if we had considered additional features, both local (such as the status of the lens) and systemic, probably our model would have been found more additional predictors of failure.

To conclude, our ML algorithm proposes a path based on some meaningful ocular surface surgical-related variables, that may guide the clinician in the pre-surgical estimation of the risk of failure. When considering the entire CT algorithm, the results of our study encourage surgeons to investigate the ocular surface before FS with imaging platforms, and to consider as the most crucial features the structural characteristics of the conjunctival stroma. Particular attention should be paid to patients presenting a thick and highly reflective stroma at AS-OCT, especially in younger cases. This information could permit to adopt the most appropriate surgical procedure for each patient and in implement personalized pre-, intra-, and post-operative treatment plans to improve the outcomes. When trying to translate these results into clinical practice, patients presenting unfavorable pre-operative conjunctival stromal features should be timely and accurately prepared to surgery, with the aim to contain the GTOSD and reduce the risk of failure^3^. This may imply the modulation of the IOP lowering therapy, according to the patient risk profile (removal of preservatives, suspension of the most irritating IOP lowering active compound), the use of preservative-free topical steroids and lubricants, and the eyelid status improvement.

## Methods

### Patients’ enrollment and demographic features

This prospective, observational multicenter study was carried out at the Universities of Chieti-Pescara, Pisa, and Milan (Italy). One hundred and two patients, candidates to undergo trabeculectomy for uncontrolled glaucoma, were consecutively enrolled at the Glaucoma Centers of the three research Units from January 2020 to March 2021. All patients provided written informed consent prior to enrolment, after an explanation of the purpose and possible consequences of the study. The institutional review board (IRB) of the Department of Medicine and Ageing Science (D.M.S.I. IRB) of the University G. d’Annunzio of Chieti-Pescara (Chieti, Italy) approved this study (N. 319-22), which adhered to the tenets of the Declaration of Helsinki.

Inclusion criteria were the following: age ≥ 18 years, primary open-angle or pseudo-exfoliative glaucoma, uncontrolled IOP (mean diurnal value > 21 mmHg) under maximal tolerated topical medical therapy (without oral acetazolamide). If both eyes were uncontrolled with medications and required surgery, the eye with the higher IOP or the more advanced perimetric stage (Glaucoma Staging System 2) was included^20^. Exclusion criteria were a diagnosis of secondary glaucoma (except pseudo-exfoliative or pigmentary glaucoma), history of any other ocular disease including ocular surface disease, previous ocular surgery (excluding cataract in the last 3 months), systemic or topical therapies in the last 6 months potentially affecting the ocular surface, contact lens wear, and pregnancy. Topical IOP lowering therapy had to remain unmodified until the day of surgery. Patients scheduled to undergo combined cataract and filtration surgery were excluded.

### Diagnostic procedures

At baseline (three days before surgery) patients were evaluated with the determination of the best corrected visual acuity and the IOP values (Goldmann applanation tonometry), and with the assessment of the posterior and anterior segment conditions, reserving particular attention to the site planned for surgery.

#### Ocular surface clinical tests

A questionnaire and clinical tests were performed to evaluate the symptoms and signs indicating GTOSD. The ocular surface disease index (OSDI) questionnaire, to obtain an OSDI score, was administered immediately before clinical tests, to evaluate symptoms related to GTOSD.

Afterwards, clinical tests were performed to assess signs of GTOSD, in the order suggested by the DEWS guidelines: break up time (BUT), corneal fluoresceine staining (CFS), and Schirmer test I (STI) without topical anesthesia (30 min after BUT and CFS)^21^. BUT was recorded as the average of three consecutive measurements. STI result was expressed as the length of the strip that was wet after 5 min. CFS was evaluated with 1% sodium fluorescein and scored 0 to 3 according to the Oxford grading scale^22^. Finally, since the meibum expressibility (MGE) is an easily determinable indicator of meibomian gland (MG) function, a digital pressure to the glands along the length of the eyelid, through the skin surface of the eyelid was applied through the skin surface of the eyelid. MGE was graded as follows: (1, light; 2, moderate; 3, heavy pressure)^23^.

#### Evaluation of the surgical site

A single experienced ophthalmologist per center (LB, GC and MS) performed pre-operative bulbar photographs, using a same slit lamp model (SL 9900 ELITE slit lamp (CSO, Costruzione Strumenti Oftalmici, Firenze, Italy), and similar settings. These settings required controlled room conditions (closed windows and room lights off), magnification of 10 × at slit lamp, white diffuse light to avoid undesired reflections or overexposed images, and correct patient position. Patients had to be seated comfortably, with the chin and the head tightly adherent to the rests. To further ensure the head position was optimal, photographers were recommended to line up the notch present on the headrest with the outer canthus of the eye. Finally, excellent and focused visualization of the surgical site were mandatory.

Since the correct exposure of the surgical site facilitates the surgeon during the different steps of the procedure, thus permitting to finalize the technical execution of filtration surgery at best, we pre-operatively measured the area of exposure of the entire upper bulbar conjunctiva and the perpendicular linear distance between limbus (at twelve o’clock) and eyelid margin. With the patient in downward gaze and gently elevating the upper eyelid, a set of photographs were performed at slit lamp to the entire conjunctival field available for the surgical procedure. The Image J software (https://imagej.nih.gov/ij/) was used to measure the entire area of exposure of the bulbar conjunctiva (AEC), and the limbus eyelid margin distance (LED).

**Figure 4:**
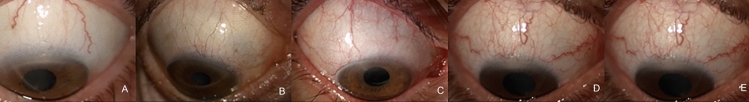
Grading of the conjunctival hyperemia according to the Mandell Classification. A. Mild bulbar conjunctival hyperemia (grade 1.2). B. Moderate bulbar conjunctival hyperemia (grade 2.2). C. Moderate circumcorneal conjunctival injection (grade 2.3). D. Severe conjunctival hyperemia (grade 3.2). E. Severe circumcorneal injection and diffuse vasodilatation (grade3.3).

Moreover, because ocular redness can be considered a surrogate indicator of the ocular surface inflammation and, therefore, of GTOSD, slit lamp photographs were also used to assess the upper bulbar conjunctival hyperemia (BCH). The Mandell classification scale, which grades conjunctival and circumcorneal hyperemia on a 0–3.3 scale of severity, was used for this purpose (Fig. 4)^24^.

#### Anterior segment-optical coherence tomography (AS-OCT) of the superior bulbar conjunctiva

To image the ocular surface, patients underwent AS-OCT (Cirrus HD-OCT 5000 (Carl Zeiss Meditec Inc., Dublin, CA)) of the superior bulbar conjunctiva, at the site planned for FS, immediately before surgery. Since the optical interferometry system of the OCT generates a log reflectivity profile, the device permits to differentiate and investigate the anatomical features of each conjunctival layer. The ASOCT assessment of the superior bulbar conjunctiva was previously described^12^.

A 4 × 4 mm area of the superior bulbar conjunctiva, centered at the 12 o'clock meridian and corresponding to the site of filtration bleb formation, was marked using methylene blue and analyzed with the Anterior Segment 5 Line Raster (3 mm long, separated by 250 μm) scanning protocol. To investigate at best the entire area of interest, three consecutive scans (limbal, central, posterior) were performed; the individual line with the greatest clarity of detail for each of the three scans was selected for the analysis by the high-definition image analysis protocol.

Four ASOCT parameters were considered: epithelial and stromal bulbar thickness (CET and CST), and the epithelial and stromal conjunctival reflectivity (ECR, SCR). The thickness parameters were evaluated along the highest clarity line of the three scans, positioning the caliper where tissue landmarks were mostly defined (conjunctiva from the sclera, and epithelium from stroma within the conjunctiva). Three full, epithelial, and stromal thickness measurements were performed for each scan, and the mean value of three measurements was considered; finally, the mean value of the three scans was used for the analysis. Landmarks were identified by differences in brightness between tissues and by looking for visual clues that were indicative of histologic changes, such as empty spaces^25^. As previously described, full-thickness CR was evaluated by using the Image J software (http://imagej.nih.gov/ij/) and graded 0–3 according to the grey value^13^. In this grading scale 0 was indicative of a normal reflectivity (< 120.00), 1 was indicative of mild reflectivity (120.01 to 160.00), whereas 2 (160.01 to 180.00) and 3 (> 180.01) were indicative of moderate and high reflectivity, respectively. As for thickness parameters, the highest quality line of each scan was selected to calculate the CR, and the mean reflectivity value of the three scans was used for the analysis. In each of the three center involved in the enrollment, one experienced operator performed the OCT examinations (LB, GC, MS) and selected the images, which were evaluated by a second operator (RM, CP, PN); all the operators were masked for the patient history. The intra-class correlation coefficient (ICC) was used to assess agreement between the observers for OCT examinations. ICC is the ratio of the inter-subject component of discrepancy to the total discrepancy; better repeatability is obtained with a high ratio.

### Surgical procedure

Surgical procedures were performed within a period of approximately 14–18 months from the start of enrollment. A single surgeon for each center, with at least a decennial and consolidate expertise in filtration procedures for glaucoma (LM, MF, PN), performed all trabeculectomies adopting the same standard procedure. Patients underwent a 0.02% mitomycin-C (MMC) (3 min, in soaked sponges) augmented trabeculectomy, which required the creation of a fornix-based conjunctival flap, and a 4 × 4 mm, 300 µm thick scleral flap. Scleral and conjunctival flaps were both sutured down with 10-0 nylon sutures. The post-surgical therapy required the use of preservative-free dexamethasone 0.15% eye drops six times daily, tapered in 16–20 weeks, and topical preservative-free antibiotics for 2 weeks (levofloxacin 5 mg/mL). Post-operative bleb management procedures (laser suture lysis, needling, and the use of MMC) were performed when needed. Patients underwent weekly clinical controls in the first month after surgery, then monthly for the following three months, and finally at the sixth and twelfth month. Trabeculectomy was considered successful when baseline IOP reduced by 30%, with values ≤ 18 mmHg with or without the use of IOP lowering medications, at the 12th month after surgery. Two outcome classes were defined: success (C), with or without the use of IOP lowering medications, and failure (F). The main outcome of the study was to provide a classification rule for the surgical outcome in patients undergoing trabeculectomy, with a CT algorithm.

### Statistical analysis

Descriptive statistical analyses were performed to assess that the two groups (successes and failures) did not have significant differences at the baseline, and the null hypothesis was tested for each variable. Therefore, for each outcome class, continuous variables are presented as median (q1 = first quartil; q3 = third quartil) and categorical as count (n) and percentage (%). Shapiro–Wilk’s test was performed to evaluate departures from a normal distribution for each continuous variable. The Mann–Whitney U test was computed to compare continuous clinical variables, whereas chi-square tests were used to compare categorical variables between successes and failures. Univariate logistic regression models were applied to determine the study variables predictive of the surgical outcome. The results of the models were expressed as an odds ratio (OR) and a relative 95% confidence interval (95% CI). A CART was grown using binary recursive partitioning to predict the surgical outcome, to construct a decision rule, and to catch not a linear relationship between predictive factors and outcome. Recursive partitioning is a statistical tool used to separate a group into 2 subgroups, repeatedly, given some risk factors of interest^26^. In addition, the CT-growing method attempts to maximize within-node homogeneity, and the Gini coefficient was selected to measure impurity at the split. CT analysis also produces a VIRthat reflects the relative importance of each independent variable to the construction of the final tree (calculated as the change in model-predicted values per change in the independent variable’s value), regardless of whether the variable is used to split a parent node. Furthermore, the pruning procedure was performed in the CT procedure to reduce overfitting. The VIR is therefore a powerful tool for measuring and comparing the overall influence of predictor variables on the outcome of interest and provides a more complete picture than the decision tree alone can convey by accounting for variable masking^27,28^. Predictive accuracies of classification trees were then evaluated using the ROC curve and AUC. An AUC greater than 0.9 indicated excellent diagnostic efficacy, between 0.7 and 0.9 good diagnostic efficacy, and between 0.5 and 0.7 a poor diagnostic efficacy. AUC of no more than 0.5 indicated the lack of a diagnostic value of the marker using the method suggested by DeLong et al.^29^. All statistical analyses were performed using R Statistical Software (version 3.5.3; R Foundation for Statistical Computing, Vienna, Austria). All tests were two-tailed, and a p-value ≤ 0.05 was considered indicative of a statistically significant association.

## Data Availability

The datasets generated during and/or analyzed during the current study are available from the corresponding author (L.A.) on reasonable request.
